# The LPS *O*-Antigen in Photosynthetic *Bradyrhizobium* Strains Is Dispensable for the Establishment of a Successful Symbiosis with *Aeschynomene* Legumes

**DOI:** 10.1371/journal.pone.0148884

**Published:** 2016-02-05

**Authors:** Nicolas Busset, Antonia De Felice, Clémence Chaintreuil, Djamel Gully, Joël Fardoux, Sana Romdhane, Antonio Molinaro, Alba Silipo, Eric Giraud

**Affiliations:** 1 IRD, Laboratoire des Symbioses Tropicales et Méditerranéennes, UMR IRD/SupAgro/INRA/UM2/CIRAD, Campus International de Baillarguet, TA A-82/J, 34398 Montpellier Cedex 5, France; 2 Dipartimento di Scienze Chimiche, Complesso Universitario Monte Sant’Angelo, Università di Napoli Federico II, Via Cintia 4, I-80126, Napoli, Italy; Estacion Experimental del Zaidin - CSIC, SPAIN

## Abstract

The photosynthetic bradyrhizobia are able to use a Nod-factor independent process to induce nitrogen-fixing nodules on some semi-aquatic *Aeschynomene* species. These bacteria display a unique LPS *O*-antigen composed of a new sugar, the bradyrhizose that is regarded as a key symbiotic factor due to its non-immunogenic character. In this study, to check this hypothesis, we isolated mutants affected in the *O*-antigen synthesis by screening a transposon mutant library of the ORS285 strain for clones altered in colony morphology. Over the 10,000 mutants screened, five were selected and found to be mutated in two genes, *rfaL*, encoding for a putative *O*-antigen ligase and *gdh* encoding for a putative dTDP-glucose 4,6-dehydratase. Biochemical analysis confirmed that the LPS of these mutants completely lack the *O*-antigen region. However, no effect of the mutations could be detected on the symbiotic properties of the mutants indicating that the *O*-antigen region of photosynthetic *Bradyrhizobium* strains is not required for the establishment of symbiosis with *Aeschynomene*.

## Introduction

The interaction between legumes and rhizobia lead to the formation of symbiotic organs, called nodules, in which the bacteria fix nitrogen to the plant’s benefit. This symbiosis relies on an exchange of diffusible signal molecules between the two partners [1]. On one side, the plant exude in the soil flavonoid compounds that induce the expression of bacterial *nod* genes leading to the synthesis and secretion of lipochitooligosaccharides, named Nod factors (NFs). In return, the perception of NFs by the plant triggers programs that govern nodule organogenesis and bacterial infection. This molecular dialogue is encountered in most of the rhizobium/legume symbiotic systems but exceptions exist among some tropical legume species of the *Aeschynomene* genus such as *A*. *indica* and *A*. *evenia* that are nodulated by photosynthetic *Bradyrhizobium* strains (ORS278 and BTAi1) lacking the canonical *nodABC* genes required for NFs synthesis [2, 3]. In fact, the absence of *nod* genes is not a general rule among photosynthetic bradyrhizobia, some strains such as ORS285 do contain the canonical *nodABC* genes and display a broader host range that extent to all stem-nodulating *Aeschynomene* species including *A*. *afraspera*. The mutation of the *nod* genes in ORS285 strain aborts its capacity to nodulate *A*. *afraspera* but has no effect on its ability to nodulate *A*. *indica* or *A*. *evenia* [2]. This indicates first, that at least two symbiotic processes exist among the *Aeschynomene* species that differ by the requirement or not of NFs and second, that the ORS285 strain can use one or the other strategy depending on the host plant [4].

To progress in the understanding of the NF-independent symbiotic process, a large-scale transposon mutagenesis was performed on ORS278 strain to identify bacterial genes important for symbiosis [5]. Among the 25,000 clones tested on *A*. *indica* plant, more than one hundred were found affected in nodule development (Ndv- mutants). However, none of them displayed a strict nod minus phenotype suggesting functional redundancy or an essential role of symbiotic genes in bacterial survival. Since a large proportion of the Ndv- mutants were found altered in purine biosynthesis genes, it was proposed that bacterial cytokinins, which are adenosine derivatives, might constitute a key signal triggering nodule organogenesis [2, 5]. However, this hypothesis was rejected by showing that a cytokinin minus mutant of ORS285 strain is still able to nodulate *A*. *indica* [6]. The molecular mechanism of the NF-independent symbiosis remains therefore to be disclosed.

The lipopolysaccharide (LPS) is the major component of the outer membrane of Gram-negative bacteria and it is known in some rhizobial strains to play an important role during the interaction with the legume host [7, 8]. Among the three regions constituting the LPS, the lipid A, the core oligosaccharide and the *O*-antigen side chain, the last one that directly enters in contact with the host plant during the symbiotic interaction is the most variable among the bacteria. This structural diversity of the *O*-antigen region is supposed to modulate or suppress the plant defense reactions, in order to facilitate the establishment of the symbiosis [9]. Interestingly, it has been described that the LPS *O*-antigen of photosynthetic bradyrhizobia is a polymer built up on a unique bicyclic monosaccharide that had never been described before in nature and that was named bradyrhizose [10]. Furthermore, it has been shown that this *Bradyrhizobium O*-antigen does not trigger the innate immunity in different plant families, including the host plant *A*. *indica*. This led to the hypothesis that photosynthetic bradyrhizobia have evolved a non-immunogenic *O*-antigen structure to avoid the induction of the plant immune system in order to facilitate the establishment of the symbiosis [10].

In this study, to check this last hypothesis, we isolated ORS285 mutants affected in *O*-antigen synthesis by screening a transposon mutant library. Two selected mutants displaying a LPS lacking the complete *O*-antigen region were further analyzed for their ability to interact symbiotically with NF-dependent and NF-independent *Aeschynomene* species.

## Materials and Methods

### Bacterial strains and growth conditions

*Bradyrhizobium* strains ORS285 and ORS278 were isolated respectively from nodules of *Aeschynomene afraspera* and *A*. *sensitiva* [11]. These strains and their derivatives mutants in *rfaL* and *gdh* genes were grown at 34°C in yeast extract mannitol (YM) medium [12]. *Escherichia coli* strains were grown in Luria-Bertani medium (LB) at 37°C. When required, the media were supplemented with kanamycin (100 μg ml^-1^) for the *rfal* and *gdh* mutants and *E*. *coli* strains and a mixture of kanamycin (100 μg ml^-1^) and nalidixic acid (35 μg ml^-1^) for the selection of the *rfal* and *gdh* mutants.

### Transposon mutagenesis and colony selection

Transposon insertions in the ORS285 genome were generated by a biparental mating with ORS285 WT and *E*. *coli* sp. strain BW20767 containing the plasmid pCRS487 harboring the minitransposon mTn5-GNm [13] according to the protocol previously described [14]. The clones were picked and arrayed into 96-well plates, each plates containing 120 μl of YM medium with selective antibiotics. Plates were incubated for 7 days at 34°C under agitation (100 rpm). After addition of glycerol (50%) at 40 μl/well, the plates were stored at -80°C. To select clones affected in *O*-antigen synthesis, each 96-well plate of the library was inoculated onto solid media thanks to a 96-pin lid. The solid plates sealed by adhesive tape and incubated for 7 days at 37°C were then examined by eyes to identify clones displaying colony morphological alterations in comparison to the WT colonies. The selected clones were further characterized by identifying the Tn5 insertion site.

### Tn5 transposon insertion sites determination

Transposon insertion location was determined by a two stage semi-degenerative PCR and sequencing protocol according to Jacobs and associates [15]. The precise location of the Tn5 insertions in the ORS285 genome (GCA_000239755.2) was determined via a BLASTN search using the obtained sequences as query.

### LPS structure analysis

The lipopolysaccharide was extracted from the freeze-dried bacterial cells (6 g) with 1:1 hot phenol–water mixture (100 mL). The two phases were collected, dialyzed and freeze-dried. For further purification, the water phase underwent enzymatic treatment with DNase and RNase at 37°C during 14h and Proteinase K at 56°C during 14h under magnetic stirring. The recovered water phase material was ultracentrifuged at 4°C and the crude LPS obtained analyzed by SDS gel electrophoresis on a 13.5% (w/v) polyacrylamide gel. Monosaccharides were identified by GC–MS analysis of their acetylated methyl glycosides [16]. The temperature program for GC-MS analysis was isothermal at 150°C for 3 min, followed by a 5°C/min gradient up to 320°C.

### Construction of *O*-antigen mutants

Standard molecular biology techniques were used for all cloning work. All primers used for cloning of DNA fragments are listed in Table 1. For the construction of *Bradyrhizobium* strain ORS285 mutants in the gene *rfaL* (BRAO285v1_100055) and *gdh* (BRAO285v1_100023) and the *Bradyrhizobium* strain ORS278 mutant in the gene *rfaL* (BRADO5200), 300 to 400 base pairs (bp) internal fragments were amplified by PCR and cloned into the plasmid pVO155-npt2-GFP [17]. The resulting plasmids were then transferred into *E*. *coli* S17-1 strain to introduce the construction in ORS285 or ORS278 strain by mating as previously described [18].

**Table 1:** Primers used in this study.

Primers	Sequences	Relevant characteristics
285.rfal.int.f/285.rfal.int.r	CGTGGC**CTCGAG**CCGTGGACGTCGTGGCGATTC/ GACAT**TCTAGA**CATCAGCACGCAGGAGGCGAGAAAG	Cloning of BRAO285v1_100055 fragment of ORS285 in pVO155-npt2-gfp after digestion of the PCR product by XhoI/XbaI
285.gdh.int.f/285.gdh.int.r	GACGAG**CTCGAG**GACGCGTTCCTGCCCTACAAATG/ TGGTA**TCTAGA**GAAATAGGTGCGCAGGCTCATATC	Cloning of BRAO285v1_100023 fragment of ORS285 in pVO155-npt2-gfp after digestion of the PCR product by XhoI/XbaI
278.rfal.int.f/278.rfal.int.r	GGCGTC**GTCGAC**CTGATCACGATGGTGCCGTTTCTC/ GAGAATT**TCTAGA**CCTGGTCGATATAGTTCTTCAC	Cloning of BRADO5200 fragment of ORS278 in pVO155-npt2-gfp after digestion of the PCR product by SalI/XbaI

### Disk diffusion assays and NaCl resistance assay

A 4-day-old culture of ORS285 strain and its derivative mutants grown in YM was washed and adjusted to reach an optical density of one at 600 nm. Bacterial suspensions (2 ml) were then mixed with 100 ml of 42°C pre-warmed YM soft agar (0.8% agar) and 5 ml portions of this mixture were poured on YM plates. Filter disks were placed at the center of the plates, and aliquots (5 μl) of 5.5 M H_2_O_2_, 2 M HCl or sodium dodecyl sulfate (SDS) (10% w/v), were deposited on the disks. The diameters of growth inhibition areas were measured after incubation at 37°C for 5 days. Disk diffusion assays were performed in triplicates at least two independent times. For NaCl resistance assay, the same bacterial suspensions were used to inoculate YM liquid medium containing various concentrations of NaCl. The bacterial growth was monitored by following the increase in absorbance at 600 nm over time. The experiment was done in duplicates.

### Antibiotic resistance

The MIC of polymyxin B was determined by the E-test method using disk diffusion assay as described above. Strips containing a gradient of polymyxin B ranging from 0.064–1024 μg/mL (Biomérieux, Marcy-l’étoile, France) were placed in the center of plates, which were incubated at 37°C for 7 days before recording the results. The experiment was done in duplicates.

### Plant cultivations and symbiotic analysis

*Aeschynomene* seeds from *A*. *evenia* LSTM 19, *A*. *indica* LSTM 21 and *A*. *afraspera* LSTM 1 were produced in our greenhouse in LSTM and are available upon request. These seeds of surface sterilized by immersion in sulfuric acid (96%) under shaking during 40 minutes for *A*. *evenia* and *A*. *indica* or 45 minutes for *A*. *afraspera*. Seeds were abundantly washed with sterile distilled water and incubated overnight in sterile water. Seeds were then transferred for one day at 37°C in the darkness on 0.8% agar plates for germination. Plantlets were transferred on the top of test tubes covered by aluminum paper for hydroponic culture in buffered nodulation medium (BNM) [19]. Plants were grown in a 28°C growth chamber with a 16-h light and 8-h dark regime and 70% humidity. Seven days after transfer, each seedling was inoculated with one milliliter of cell suspension resulting from a 5 day-old bacterial culture washed in BNM and adjusted to reach an optical density of one at 600 nm. For nodulation and nitrogen fixation assay, ten plants per condition were taken at 14 days-post-inoculation (dpi) to count the number of nodules on the roots and to analyze the nitrogenase activity by acetylene reduction assay (ARA) as previously described [5].

### Cytological analyses and microscopy

Cytological analyses were done on 5–10 nodules originating from 3 different plants for each condition using the protocol described by Bonaldi et al [4]. Confocal microscopy observations were carried out using a confocal laser-scanning microscope (Carl Zeiss LSM 700; Jena, Germany). Calcofluor was excited at 405 nm with emission signal collection at 405 to 470 nm. For GFP, an excitation wavelength of 488 nm was used with emission signal collection at 490 to 522 nm. Images were obtained using the ZEN 2008 software (Zeiss).

## Results

### Identification of *O*-antigen mutants

**Fig 1:**
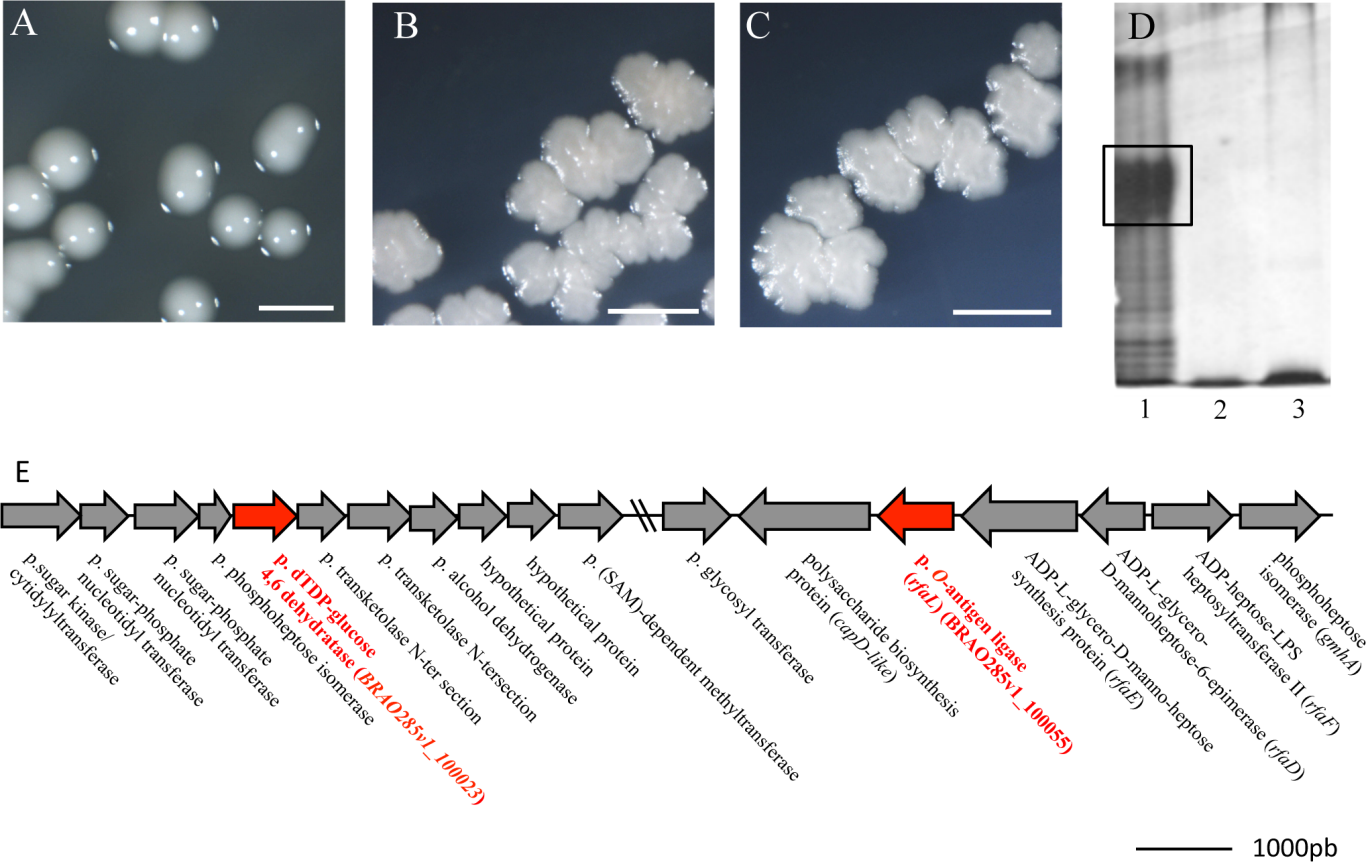
Identification of ORS285 mutants affected in the *O*-antigen synthesis. (**A**-**C)** Morphological aspect of the colonies of ORS285 (A), and two clones for which the Tn5 transposon is inserted in the CDS BRAO285v1_100023 (B) or BRAO285v1_100055 (C); scale bars, 5mm. (**D**) SDS-PAGE gel scan of the LPS of ORS285 (1), Tn5 mutant in BRAO285v1_100023 (2) and Tn5 mutant in BRAO285v1_100055 (3) mutants. The black box shows the canonical high molecular weight band of ORS285 LPS (with the *O*-antigen). All mutant strains displayed LPS lacking the complete *O*-antigen region with low molecular weight. (**E**) Genomic context of the two CDS, BRAO285v1_100023 and BRAO285v1_100055. The distance between the two CDS is about 44 kb. p., putative.

The genes involved in bradyrhizose synthesis are unknown. To progress in their identification and get mutants affected in the *O*-antigen synthesis, we screened a Tn5 mutant library of ORS285 strain for clones displaying colony morphology modifications. Indeed, it is reported that alteration of the *O*-antigen region can lead to rough or mucous colonies [8]. Over the 10,000 mutants tested, five clones displayed colony morphologies different to the WT strain with an irregular borderline and a rough aspect (Fig 1A–1C).

The identification of the Tn5 insertion sites permitted to identify two genes in which the five clones were found mutated. The first one (BRAO285v1_100055) encodes a putative *O*-antigen ligase that is involved in the ligation of the *O*-antigen to the lipid A-core oligosaccharide [20]. There are several arguments to sustain the functional prediction of this CDS: i) the corresponding protein of 427 AA contains a Wzy_C domain (PFAM04932) which is characteristic of *O*-antigen ligases, ii) the protein displays a high level of identity (63%) with the CDS Bll5926 of *B*. *japonicum* which has been recently shown to be required for the *O*-antigen synthesis and which has been annotated as *rfaL* [21] and iii) the gene is found in the same genomic context than the *rfaL* gene of *E*. *coli* with the presence at the vicinity of *rfaD*, *rfaE* and *rfaF* homologs (Fig 1E).

The second one (BRAO285v1_100023) encodes a putative dTDP-glucose 4,6-dehydratase. This gene is found in a cluster of eleven genes encoding for most of them sugar modification enzymes. Interestingly, this gene cluster is located at only 40-kb from the *rfaL* gene previously identified suggesting that this DNA region is most probably dedicated to the LPS biosynthesis (Fig 1E).

Hereafter, we renamed the CDS BRAO285v1_100055 to *rfaL* and the CDS BRAO285v1_100023 to *gdh* for **g**lucose **d**e**h**ydratase. It is to highlight that these two genes belonging to operons it cannot be excluded the possibility that the mutants' phenotype was due to polar effects. Nevertheless, these mutants remain useful to examine the symbiotic role of the *O*-antigen.

### The *rfaL* and *gdh* mutants display a LPS lacking the *O*-antigen region

**Fig 2:**
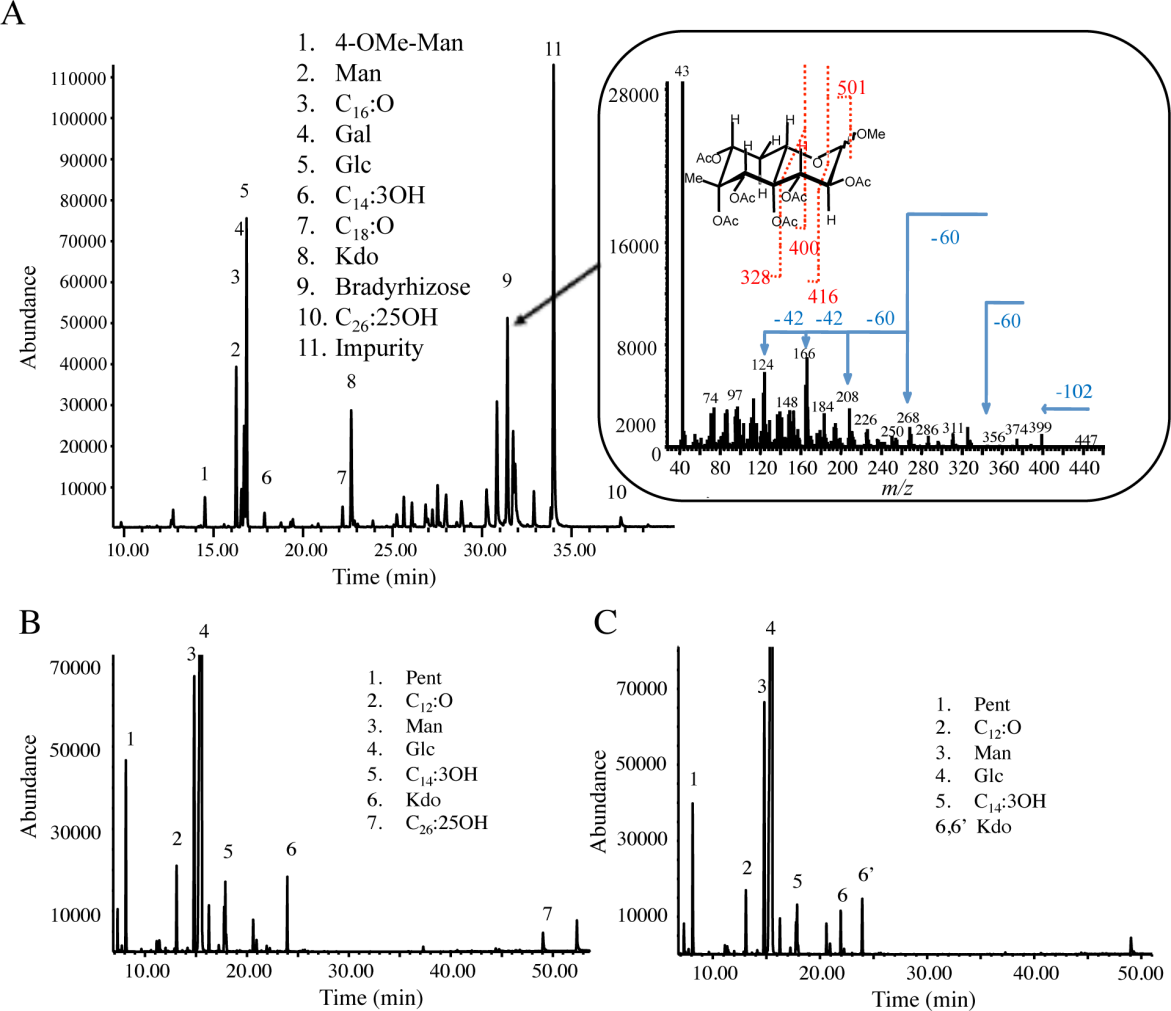
The *rfaL* and *gdh* mutants of ORS285 display a LPS lacking the *O*-antigen region. (**A**-**C**) GC-MS chromatogram of LPS from ORS285 (A), *rfal* mutant (B) and *gdh* mutant(C). (A) In the inset the compositional analysis is shown and at about 30 min retention time all the bradyrhizose peaks are also shown. In the section on the right the mass spectrum of ion peak 9 with fragmentation pattern of bradyrhizose is visible as well. Both mutant LPS do not display the typical ion peaks of bradyrhizose at retention time around 30 min. Pent, pentose; Man, mannose; Glc, glucose; Gal, galactose; Kdo, 3-deoxy-d-manno-oct-2-ulosonic acid; C12:O, dodecanoic acid; C14:3OH, tetradecanoic acid; C16:O, hexadecanoic acid; C18:O, octadecanoic acid; C26:25OH, 25-hydroxy-hexacosanoid acid.

To confirm that the selected mutants were affected in the *O*-antigen-synthesis, we compared the LPS profile of two of them affected respectively in the *rflaL* and *gdh* genes. As shown in Fig 1D, drastic difference was observed between the two mutants and the WT-strain with notably the absence for the mutants of the major band corresponding to the *O*-antigen region in the WT. To confirm this data, the purified LPS of the two mutants was further analyzed by GC-MS. As shown in Fig 2A–2C, ion peaks belonging to bradyrhizose (ion peaks from 25 to 32 min) present in spectrum of WT LPS were not observed in the mutant LPS. Further, the compositional analysis of the core oligosaccharide revealed slight differences between wild type and mutants strains, consisting in the absence of a methylated monosaccharide and indicate that the LPS of the two selected Tn5 mutants completely lack the *O*-antigen region and only display sugar residues belonging to the core region.

**Fig 3:**
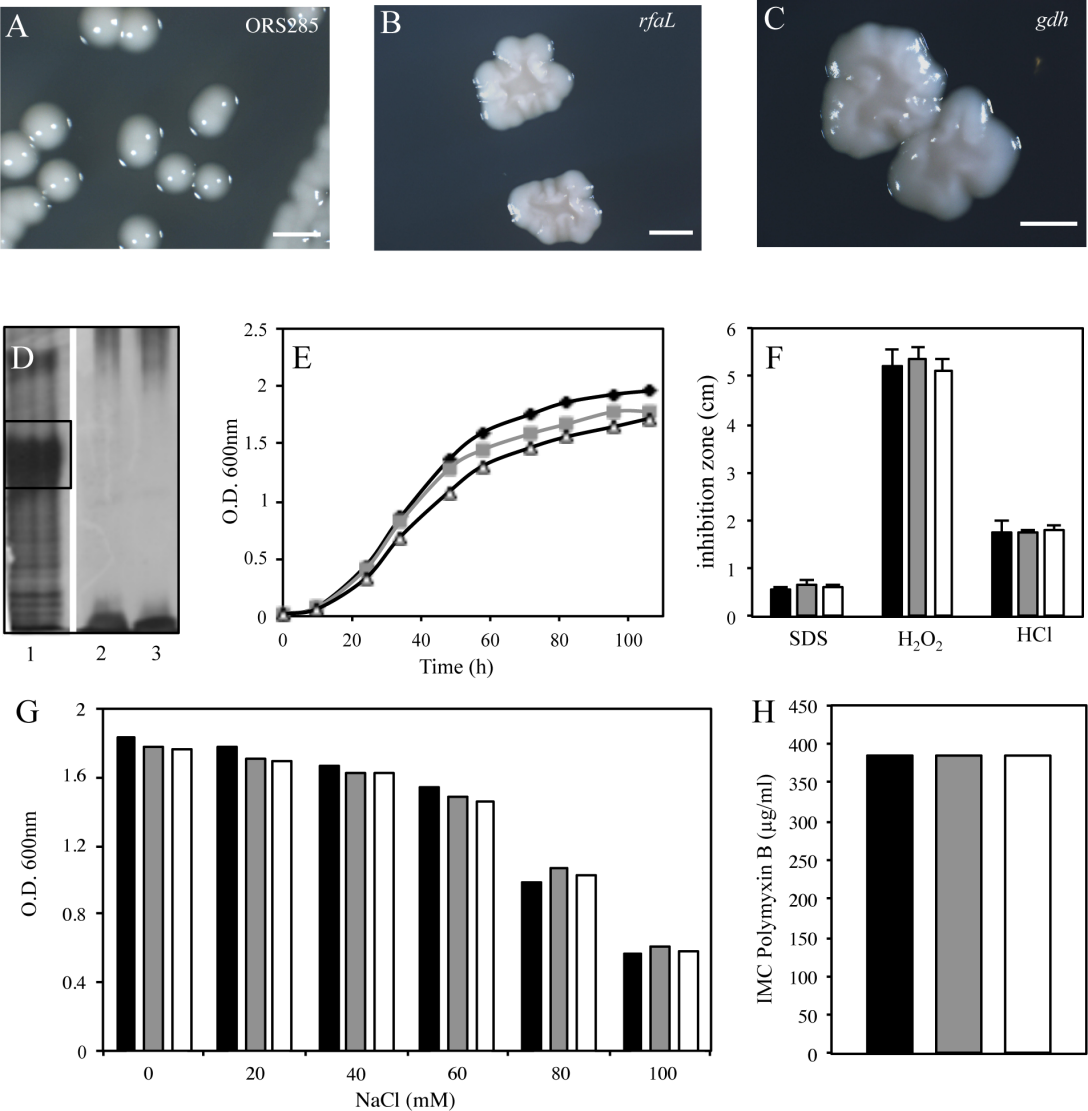
The lack of *O*-antigen has no impact on the free life of ORS285. (**A**-**C**) Colony morphotypes of ORS285 (A), *rfal* (B) and *gdh* (C) mutants; scale bars, 2mm. (**D**) SDS-PAGE gel scan of the LPS of ORS285 (1), *rfal* (2) and *gdh* (3) mutants. The black box shows the canonical high molecular weight band of ORS285 LPS (with the *O*-antigen) that is absent in the *rfal* and *gdh* mutants. (**E**) Growth of ORS285 (black), *rfal* (grey) and *gdh* (white) mutants in YM medium at 37°C. (**F**) Hydrogen peroxide (H_2_O_2_), hydrogen chloride (HCl) and sodium dodecyl sulfate (SDS) resistance of ORS285 (black bar), *rfal* (grey bar) and *gdh* (white bar) mutants, as determined by disk diffusion assays using 5 ml of 5.5M H_2_O_2_, 2N HCl or 10% of SDS. Error bars represent standard errors (n = 9); Tukey’s honestly significant difference test indicates no significant effect (*P* < 0.01). (**G**) Growth of ORS285 (black bar), *rfal* (grey bar) and *gdh* (white bar) mutants, in YM medium supplemented with various concentration of NaCl at 37°C. (**H**) Polymyxin B resistance of ORS285 (black bar), *rfal* (grey bar) and *gdh* (white bar) mutants, as determined by Etest (Etest®bioMérieux) on YM medium (performed and interpreted according to the manufacturer's procedures).

To validate that the absence of the *O*-antigen in the two selected mutants was due to the transposon insertion in the *rfaL* and *gdh* genes and not to another mutation, we constructed new insertional mutants in these two genes by using the non-replicative plasmid pVO155-npt2-GFP. This plasmid that harbors a constitutive GFP was chosen to facilitate the later analysis of the symbiotic properties of the mutants (see above). The two new mutants obtained displayed the same colony morphology phenotype than the transposon mutants (Fig 3A–3C) and analysis of their LPS profile by SDS-PAGE confirmed the absence of the *O*-antigen region (Fig 3D). Altogether these data confirm that the CDS BRAO285v1_100055 encodes for an *O*-antigen ligase (*rfaL*) and suggest that the cluster of 11 genes including *gdh* is involved in the synthesis of the *O*-antigen precursor, i.e. the bradyrhizose.

### The lack of *O*-antigen does not impact the resistance of ORS285 strain to stresses

Besides modification of the colonies aspect, it has been reported that the removal of the *O*-antigen region could alter the sensitivity of some mutants to various stresses [22, 23]. In particular in *B*. *japoncium*, the *rfaL* mutation slightly increases the sensitivity of the mutant to osmotic and oxidative stresses [21]. This higher sensitivity can compromise the success of the symbiotic interaction because all along the process the bacteria has to cope with various stressful conditions such as acidic pH, high osmolarity, reactive oxygen species, and peptide antibiotics [24, 25].

To determine whether the lack of *O*-antigen has an effect on the ORS285 strain cultivated under free-living conditions, we first analyzed the growth on YM medium of the *gdh* and *rfaL* mutants obtained after pVO155 insertion. As shown in Fig 3E, the two mutants displayed a similar growth than the WT-strain. We also analyzed the ability of the mutants to cope with various stressors, acid (HCl), oxidant (H_2_O_2_) and detergent (Sodium dodecyl sulfate) using disk diffusion assays and to saline/osmotic stress by comparing bacterial growth rates in the presence of increasing concentrations of NaCl. However, similarly, no significant difference was observed between the two mutants and the WT-strain (Fig 3F and 3G). Likewise, the two mutants display the same resistance than the WT to the antimicrobial peptide, polymyxin B (Fig 3H). Altogether, these data suggest that the *O*-antigen region does not play an important role in the ability of the ORS285 strain to cope with various stressful conditions.

### The LPS *O*-antigen mutants are not affected in their symbiotic properties with *Aeschynomene* legumes

**Fig 4:**
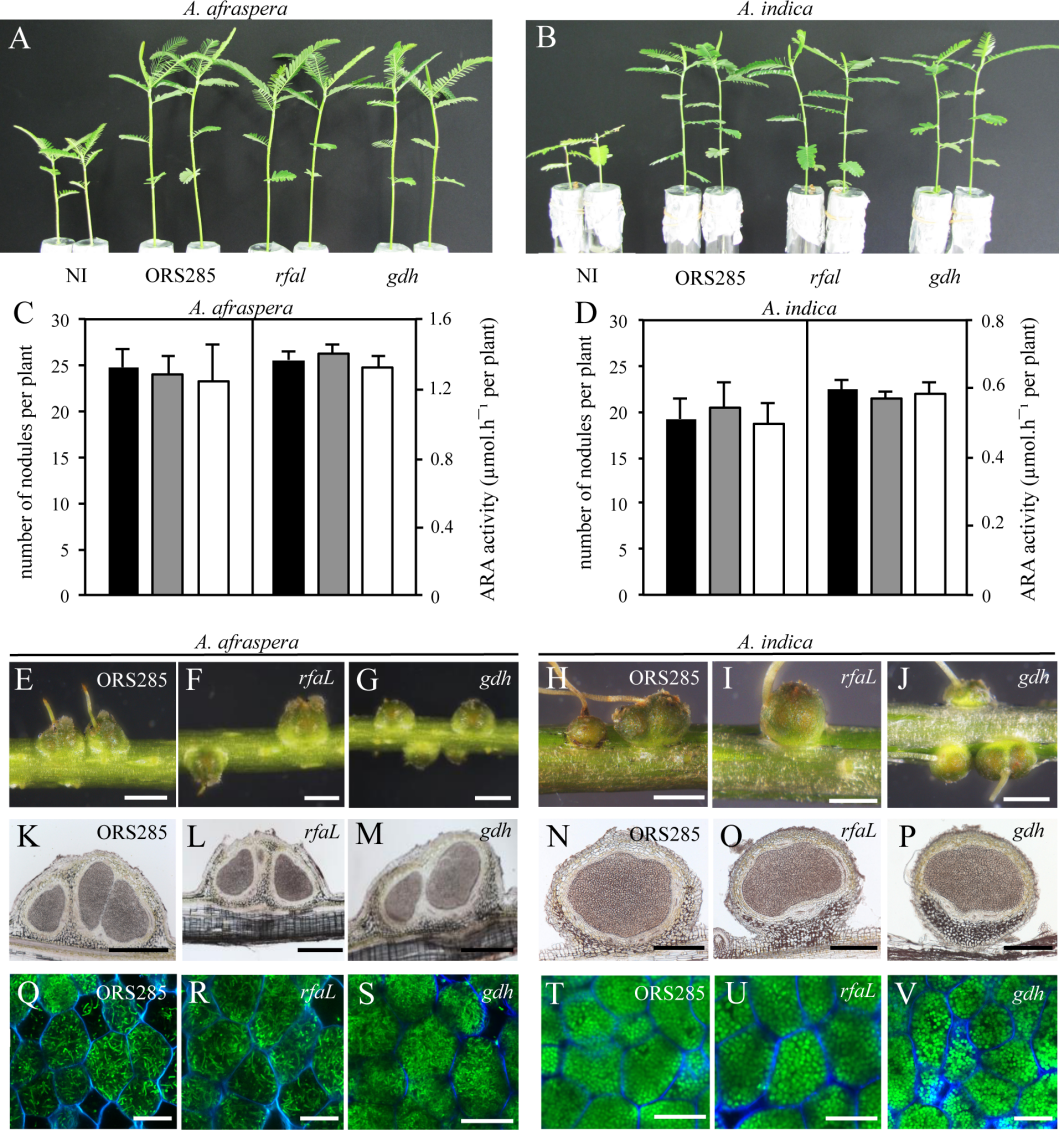
*O*-antigen minus mutants of ORS285 are not affected in their symbiotic properties with *Aeschynomene* legumes. (**A**, **B**) Comparison of the growth of *A*. *afraspera* (A) and *A*. *indica* (B) (aerial part), noninoculated (N.I.) or inoculated with ORS285, *rfal* or *gdh* mutants. (**C**, **D**) Quantification of acetylene reduction activity (ARA) and number of nodules per plant inoculated with ORS285 (black bars), *rfal* (grey bars) or *gdh* (white bars) mutants in *A*. *afraspera* (C) and *A*. *indica* (D). Error bars represent standard deviations (n = 10); Tukey’s honestly significant difference test indicates no significant effect(*P* < 0.01). (**E**-**J**) Whole roots of *A*. *afraspera* (E-G) and *A*. *indica* (H-J) inoculed with ORS285 (E, H), *rfaL* (F, I) or *gdh* (G, J) mutants; scale bars, 1 mm. (**K**-**P**) Nodule thin sections of *A*. *afraspera* (K-M) and *A*. *indica* (N-P), elicited by ORS285 (K, N), *rfaL* (L, O) or *gdh* (M, P) mutants and viewed by bright-field microscopy; scale bars, 400 μm. (**Q**-**V**) Confocal microscopy observations of nodules from *A*. *afraspera* (Q-S) and *A*. *indica* (T-V) elicited by ORS285 (Q, T), *rfal* (R, U) and *gdh* (S, V) mutants; scale bars, 20 μm.

To determine whether the *O*-antigen plays a symbiotic role in photosynthetic bradyrhizobia, we inoculated the ORS285 strain and the *rfaL* and *gdh* mutants on both *A*. *afraspera* and *A*. *indica*. These two host plants were selected because they differ in their requirement of NFs to initiate the symbiosis with ORS285 strain [4]. Observations done at 14 days post inoculation (dpi) which corresponds to a lapse of time classically used to estimate symbiotic performance of mutants [5], did not reveal difference in the growth of the two *Aeschynomene* plants inoculated with the WT strain or the two mutants (Fig 4A and 4B). This is correlated with a comparable number of nodules elicited per plants and a similar nitrogenase activity per plant estimated by acetylene reduction assay (ARA) (Fig 4C and 4D). To go further in the characterization of the symbiotic properties of the two mutants, cytological analysis of the nodules were performed. The nodules elicited by the mutants looked like the WT- nodules (Fig 4E–4P), the central tissue was homogeneously infected and displayed no signs of degeneracy nor plant defense reactions indicating that the lack of the *O*-antigen do not compromise nodule development and bacterial invasion. To check that the mutants were not affected during the differentiation step, we took benefit of the GFP-tag used to construct the mutants to analyze by confocal microscopy the aspect of the bacteroids. As observed for the WT strain, the two mutants were found perfectly differentiated into elongated bacteroids in *A*. *afraspera* and spherical bacteroids in *A*. *indica* (Fig 4Q–4V). Taken together, these data indicate that the absence of the *O*-antigen do not interfere with the ability of the ORS285 strain to establish symbiosis with *Aeschynomene* plants irrespective of the symbiotic process used.

We considered also the possibility that the NFs synthetized by ORS285 strain, even if they are dispensable for the establishment of the symbiosis with *A*. *indica*, could interfere during the interaction of the bacteria with the plant and mask the role played by the *O*-antigen. To check this possibility, we constructed an *O*-antigen mutant in the strain ORS278 that lacks the canonical *nodABC* genes. This mutant was obtained by mutating the CDS BRADO5200 that displayed 95% of AA identity with RfaL of ORS285 strain. As shown in S1A and S1B Fig, the mutant formed irregular and smooth colonies and MS analysis of the extracted LPS confirmed that it lacked the *O*-antigen (S1C and S1D Fig). The mutant obtained was then tested on the two Nod-independent *Aeschynymene* species (*A*. *indica* and *A*. *evenia*) but no difference was observed with the ORS278 WT strain (S2 Fig). Altogether these data confirm that the *O*-antigen region of photosynthetic *Bradyrhizobium* strains is dispensable for the establishment of symbiosis with *Aeschynomene*.

## Discussion

Structural analysis of the LPS of photosynthetic *Bradyrhizobium* strains (BTAi1 and ORS278) has revealed two original features: i) the *O*-antigen region which consists of a unique monosaccharide, named bradyrhizose, [10], and ii) the lipid A region which displays the covalent attachment of a hopanoid molecule to a very long chain fatty acid [25]. We have recently shown that this unusual combination of hopanoid to the lipid A, named HoLA for Hopanoid Lipid A, permits to reinforce the stability and rigidity of the outer membrane and plays an important role in the free living and symbiotic states of the bacteria. In particular, a hopanoid-deficient mutant lost its ability to maintain a chronic intracellular infection [25].

In this study, we investigated whether the *O*-antigen region, which has been shown to be non-immunogenic in various plants, is important for the ability of photosynthetic *Bradyrhizobium* strains to establish a symbiosis with *Aeschynomene* plants. Using an approach without a priori, we identified two genes that are required for the synthesis or the attachment of the *O*-antigen to the LPS. The first one, *rfaL*, encodes an *O*-antigen ligase, the second one, *gdh*, encodes for a dTDP-glucose 4,6-dehydratase. Mutants affected in these two genes, which form LPS completely lacking the *O*-antigen part, do not display particular symbiotic defects. This absence of phenotype is observed for mutants of two photosynthetic *Bradyrhizobium* strains (ORS278, ORS285) and using various *Aeschynomene* species that differ by the requirement or not of NFs. This result is striking considering the crucial role that has been reported for the *O*-antigen region in other rhizobium-legume symbiosis. In particular, LPS mutants deficient in *O*-antigen in *B*. *japonicum*, *Rhizobium phaseoli*, *Rhizobium etli*, *Rhizobium leguminosarum* and *Azorhizobium caulinodans* were all reported to be drastically affected in symbiosis, either in the infection process, the nodule development or in the bacteroid differentiation step [21, 26–29]. So, why the deletion of the *O*-antigen region do not affect the ability of photosynthetic *Bradyrhizobium* strains to interact with *Aeschynomene*? Several hypotheses can be advanced. First, the core oligosaccharide that becomes in these mutants the most external part susceptible to enter directly in contact with the host cell is also non-immunogenic. The structural analysis of LPS core region in the ORS285 strain is in progress. Second, the photosynthetic bradyrhizobia are coated with other non-immunogenic surface polysaccharides, such as EPS, KPS and/or cyclic glycans that mask the LPS antigenic epitopes. They are several examples indicating that such surface polysaccharides play also an important role in the establishment of the rhizobium-legume symbiosis either by suppressing the plant immunity or by masking some surface antigens or by acting directly as a symbiotic signal [7, 9, 30]. Finally, we cannot exclude that the photosynthetic bradyrhizobia produce unknown non-Nod signal(s) that besides triggering the symbiotic process, suppress the plant innate immunity, such as proposed for Nod factors [31, 32]. Further studies will be required to better understand the molecular mechanisms permitting the establishment of the NF-independent symbiosis and to precise whether the bradyrhizose plays a specific biological function. In particular, considering that the *O*-antigen could play a more prominent role in natural conditions, it would be interesting to do competitive experiments between the WT strain and the *O*-antigen mutants and to use lower amount of inoculum.

The identification that the mutation in the *gdh* gene leads to a LPS lacking *O*-antigen is another interesting aspect of this study. This gene belongs to a gene cluster of eleven genes for which the synteny is perfectly conserved among the 6 photosynthetic *Bradyrhizobium* strains sequenced (ORS278, ORS285, BTAi1, STM3809, ORS375, *B*. *oligotrophicum*) suggesting that this gene cluster forms an operon. We could therefore not exclude the possibility that the absence of *O*-antigen in the corresponding mutant results to a polar mutation. The fact that this genes cluster encodes for several sugar modification enzymes and that an insertional mutation led to the absence of the *O*-antigen, led us to assume that this operon is dedicated to the synthesis of the *O*-antigen precursor, i.e. the bradyrhizose. Interestingly, the bradyrhizose polymer tends to adopt a compact two-fold right-handed helicoidal structure forming an hydrophobic tunnel inside the helix form [10]. This specific supramolecular architecture could be used to trap some hydrophobic molecules of interest inside this tunnel. Coupled to the fact that this polymer is non-immunogenic, some biotechnological applications could emerge from bradyrhizose. It has been recently proposed a chemical process to synthetize in 26 steps the bradyrhizose [33]. With the identification of this gene cluster, this study could open to new strategy to synthesize this sugar using biotechnical approaches.

## Supporting Information

S1 Fig The *rfaL* mutant of ORS278 displays a LPS lacking the *O*-antigen region. (**A**, **B**) Colony morphotypes of ORS278 (A) and *rfal* mutant (B); scale bars, 5mm. (**C**, **D**) GC-MS chromatogram of LPS from ORS278 (C) and *rfal* mutant (D). The mutant LPS (D) do not display O-antigen region at about 30 min retention time. Man, mannose; Glc, glucose; Gal, galactose; Hep, heptose; Kdo, 3-deoxy-d-manno-oct-2-ulosonic acid; C12:O, dodecanoic acid; C14:3OH, tetradecanoic acid; C16:O, hexadecanoic acid; C18:O, octadecanoic acid; C25:O, pentacosanoic acid; C26:25OH, 25-hydroxy-hexacosanoid acid.
(TIF)

S2 Fig The *rfaL* mutant of ORS278 is not affected in its symbiotic properties with *Aeschynomene* legumes. (**A**, **B**) Comparison of the growth of *A*. *indica* (A) and *A*. *evenia* (B) (aerial part), inoculated with ORS278 or *rfal* mutant. (**C**, **D**) Quantification of acetylene reduction activity (ARA) and number of nodules per plant inoculated with ORS278 (black bars) or *rfaL* mutant (grey bars) in *A*. *indica* (C) and *A*. *evenia* (D). Error bars represent standard deviations (n = 10); Tukey’s honestly significant difference test indicates no significant effect (*P* < 0.01). (**E**-**H**) Whole roots of *A*. *indica* (E, F) and *A*. *evenia* (G, H) inoculated by ORS278 (E, G), and *rfal* mutant (F, H); scale bars, 1 mm. (**I**-**L**) Nodule thin sections of *A*. *indica* (I, J) and *A*. *evenia* (K, L), elicited by ORS278 (I, K) or *rfaL* mutant (J, L) and viewed by bright-field microscopy; scale bars, 400 μm. (**M**-**P**) Confocal microscopy observations of nodules from *A*. *indica* (M, N) and *A*. *evenia* (O, P) elicited by ORS278 (M, O), and *rfaL* mutant (N, P); scale bars, 20 μm.
(TIF)
