# Supplementary figures and images for: The LPS O-Antigen in Photosynthetic Bradyrhizobium Strains Is Dispensable for the Establishment of a Successful Symbiosis with Aeschynomene Legumes
Source: PLoS One. 2016 Feb 5;11(2):e0148884. doi: 10.1371/journal.pone.0148884 (PMC4743980; doi:10.1371/journal.pone.0148884)

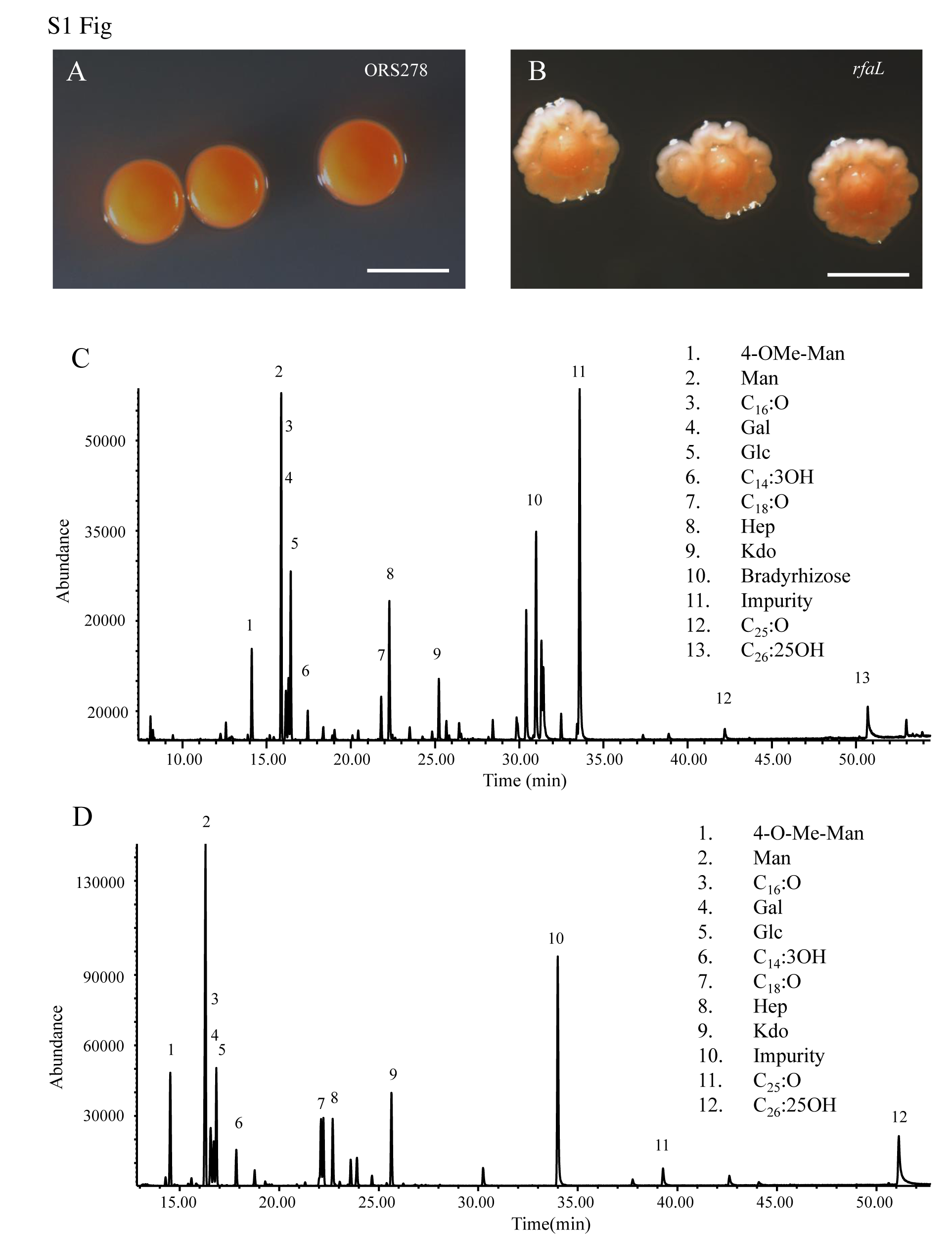

Supplement: S1 Fig — (A, B) Colony morphotypes of ORS278 (A) and rfal mutant (B); scale bars, 5mm. (C, D) GC-MS chromatogram of LPS from ORS278 (C) and rfal mutant (D). The mutant LPS (D) do not display O-antigen region at about 30 min retention time. Man, mannose; Glc, glucose; Gal, galactose; Hep, heptose; Kdo, 3-deoxy-d-manno-oct-2-ulosonic acid; C12:O, dodecanoic acid; C14:3OH, tetradecanoic acid; C16:O, hexadecanoic acid; C18:O, octadecanoic acid; C25:O, pentacosanoic acid; C26:25OH, 25-hydroxy-hexacosanoid acid. (TIF) [file pone.0148884.s001.tif]

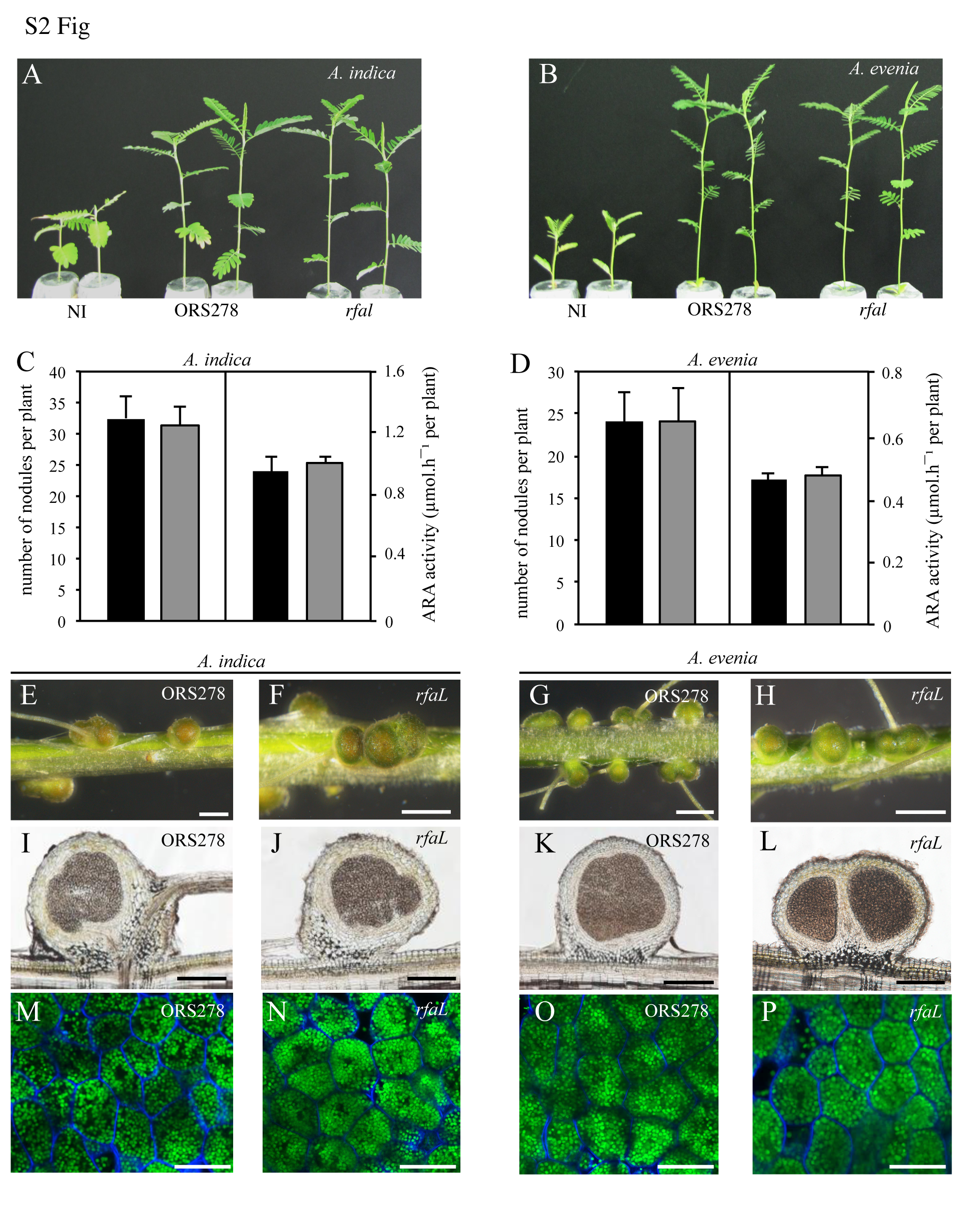

Supplement: S2 Fig — (A, B) Comparison of the growth of A. indica (A) and A. evenia (B) (aerial part), inoculated with ORS278 or rfal mutant. (C, D) Quantification of acetylene reduction activity (ARA) and number of nodules per plant inoculated with ORS278 (black bars) or rfaL mutant (grey bars) in A. indica (C) and A. evenia (D). Error bars represent standard deviations (n = 10); Tukey’s honestly significant difference test indicates no significant effect (P < 0.01). (E-H) Whole roots of A. indica (E, F) and A. evenia (G, H) inoculated by ORS278 (E, G), and rfal mutant (F, H); scale bars, 1 mm. (I-L) Nodule thin sections of A. indica (I, J) and A. evenia (K, L), elicited by ORS278 (I, K) or rfaL mutant (J, L) and viewed by bright-field microscopy; scale bars, 400 μm. (M-P) Confocal microscopy observations of nodules from A. indica (M, N) and A. evenia (O, P) elicited by ORS278 (M, O), and rfaL mutant (N, P); scale bars, 20 μm. (TIF) [file pone.0148884.s002.tif]
